# BFEX: A Toolbox for Finite Element Analysis With Fossils and Blender

**DOI:** 10.1002/ece3.71093

**Published:** 2025-03-06

**Authors:** E. Miguel Díaz de León‐Muñoz, Romain Boman, Gabriel S. Ferreira

**Affiliations:** ^1^ Fachbereich Geowissenschaften University of Tübingen Tubingen Germany; ^2^ Department of Aerospace and Mechanical Engineering, Non‐Linear Computational Mechanics (MN2L) Research Group University of Liège Liege Belgium; ^3^ Senckenberg Centre for Human Evolution and Palaeoenvironment Tubingen Germany

## Abstract

(1) Finite Element Analysis (FEA) has become a common method when studying fossils. As computer systems and tools advance, new software like Fossils is developed to make FE analyses more efficient. (2) However, the preparation of input data for analysis remains time‐consuming. In particular, surface mesh cleaning and the selection of groups of nodes for the boundary conditions (muscle forces and prescribed displacements) usually require graphical programs that are either not well adapted to this task or require an expensive license. (3) To address this, we introduce BFEX (Blender Finite Element eXporter), a Blender add‐on that simplifies model creation for FEA with Fossils. (4) This approach helps streamline the entire process, making it more user‐friendly.

## Graphical abstract

We introduce BFEX, a Blender add‐on for building Finite Elements Analysis for Fossils software. This approach streamlines the process of creating and visualizing FE models.

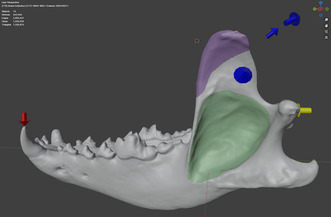

## 1 Introduction

In recent years, biomechanical studies using Finite Element Analysis (FEA) have gained popularity within the field of paleontology and biology. By simplifying complex structures into elements of simpler geometries with known mechanical properties and boundary conditions, FEA enables researchers to simulate different biological scenarios and test specific hypotheses about the behavior of such structures (Zienkiewicz and Taylor 2005; Zienkiewicz and Morice 1971; Rayfield 2007; Bright 2014). In addition to its methodological power, the increased availability of different software options is likely also related to the increased usage of FEA in those areas. Among those, many are commercial software and thus require costly licenses, such as ANSYS, Abaqus, and Strand7, which were mostly developed for engineering applications. However, free open‐source alternatives developed more specifically for biological and paleontological applications also exist, such as FEBio (Maas et al. 2012) and *Fossils*, respectively. *Fossils*, the implementation of a protocol published by (Chatar et al. 2023), provides a free and effective approach for simulating muscle‐driven biomechanical loading on bones. Despite the current limitations of *Fossils* (e.g., lack of support for multiple materials, non‐linear material properties, absence of graphical user interface to build the model), it has been shown to be a powerful tool with a better performance than other commercial software such as Strand7 (Chatar et al. 2023) In addition to its superior performance, *Fossils* offers several advantages over commercial software: (1) it is user‐friendly without the overwhelming complexity of commercial software, making it accessible for individuals without prior experience in FEA, (2) its open‐source nature allows users to modify the code to suit their needs, fostering a community of contributors to the software's development, and (3) it helps reduce research costs by providing a free alternative to commercial software.

Despite the aforementioned benefits, the lengthy process of preparing FE models, including the mesh, loads, and boundary conditions, combined with the lack of a graphical user interface and the need to use a Python script, may deter new users from adopting *Fossils* in their routine.

*Fossils* requires at least two input files to perform a FEA (for a full explanation see (Chatar et al. 2023)), the first one is a 3D model mesh. This object can be provided in STL, OBJ, or PLY format. The second file is the Python script with all the mechanical parameters needed to build and solve the FE model. Additional files are required only if the user is interested in testing intrinsic scenarios (i.e., muscle‐driven simulations) and they consist of samples of the main object representing the attachment area of the muscles in STL, OBJ, or PLY format. The Python file contains (1) the name and path of the mesh(es), (2) the X, Y, and Z coordinates of the contact point(s) (i.e., the points at which a reaction force will be calculated, e.g., the tip of a tooth), (3) the X, Y, and Z coordinates of the constraint point(s) (e.g., the temporomandibular joints). The last section of the Python file includes (4) the material properties of the bone: density, Young's modulus and Poisson's ratio. For each muscle used in the analysis, it is also necessary to provide the path of the mesh file, the force applied by that muscle, the coordinates to a focal point towards which the force will be applied, and the loading scenario (Directional Traction Model, Normal Traction Model, Uniform Traction Model, Tangential‐Traction Model or Tangential‐Plus‐Normal‐Traction Model). Since version 1.3 of *Fossils*, it is possible to define points or surfaces where a load will be applied to simulate extrinsic scenarios. The creation and preparation of these files can be time‐consuming, especially for new users.

Blender (Blender Development Team 2024) is an open‐source project focused on 3D animation, which also offers a wide variety of powerful tools for processing and modifying meshes. Blender has become a valuable tool for paleontologists and has been used in paleontology for retrodeformation and restoration of fossils (DeVries et al. 2022), for analyses of locomotion (Garwood and Dunlop 2014), and reconstruction of muscles to estimate volumes and forces (Herbst et al. 2022) and for estimating the maximum jaw gape of dinosaurs (Lautenschlager 2015).

In this context, we introduce BFEX (Blender Finite Elements Exporter), a new Blender add‐on that provides a user‐friendly interface to prepare FE models to be solved using *Fossils*. BFEX offers a set of solutions to improve the efficiency of the workflow and options that allow the user to easily build a FE model containing a mesh file and the input parameters. Currently, BFEX is supported only by Blender +4.2 versions.

## 2 Workflow for FEA With Blender and Fossils

Before running a FE analysis, proper preparation of the mesh is a prerequisite. This preparation starts with the geometry acquisition, usually through one of the most common techniques: photogrammetry, 3D surface scanning, or Computed Tomography (CT). For a review of the advantages and disadvantages of each technique, see (Sutton et al. 2016) and (Cunningham et al. 2014). Some useful guides based on open‐source software about how to generate 3D models and export meshes are available for the different techniques; for example, see (Buser et al. 2020) for processing files obtained from CT scanners and (Díaz de León Muñoz 2017) or (Zhang and Maga 2023) for processing files for photogrammetry.

The first step after importing a mesh into Blender is to check its scale and quality. In the case of the scale, it is important to keep in mind that sometimes Blender misinterprets the units of the scale of the object. In our experience, most of the time Blender will interpret millimeters as meters on models generated as STL or Obj objects. For this reason, we recommend not to change the default unit in Blender and interpret it as millimeters without making any changes. This is relevant for the correct use of force units in the FEA and the correct interpretation of the results. If necessary, the user can change the scale of the object.

Regarding the quality, the raw mesh will usually require some post‐processing, involving thorough cleaning and reconstruction. Surface meshes commonly exhibit undesired elements such as overlapping faces, holes, and non‐manifold faces, and *Fossils* will fail if there are any of those. And even if volume mesh generation does not fail, the results can be biased if the face sizes are too heterogeneous. Unlike other software such as MeshLab (Cignoni et al. 2008), Blender is a non‐destructive tool, meaning that the modifications to fix errors can be undone if the results are not satisfactory.

To generate a more homogeneous mesh, we recommend remeshing the surface of the object (*Modifiers > Add Modifier > Generate > Remesh*) to avoid large or high aspect‐ratio elements (Marcé‐Nogué et al. 2016). After remeshing, it is necessary to triangulate the surface (*Modifiers > Add Modifier > Generate > Triangulate*) because *Fossils* cannot deal with quadrangular elements. Then the mesh can be inspected using the 3D‐Print add‐on included with Blender, which can be installed in the preferences menu (*Edit > Preferences*). In the 3D‐Print add‐on, the user should check for: non‐manifold edges, bad contiguous edges, intersecting faces, zero faces, zero edges, non‐flat faces, thin faces, and sharp edges. Zero faces, zero edges, and thin faces can be eliminated by dissolving the problematic face with another contiguous face. For the non‐flat faces, it is possible to select them and press *ctrl + T* to triangulate them. Other errors may be solved using the tools in the *sculpt mode*. After completing the manual corrections, it might be necessary to remesh the object again to obtain a more homogeneous mesh to make sure the elements are not too different from one another.

If the user decides to remesh the object, some considerations have to be taken into account: generally, a higher number of faces results in a more complex and accurate geometry (other quality traits may also affect the FEA results, see (Marcé‐Nogué et al. 2015)). However, a more complex mesh requires more computing time to solve the mechanical problem. The user should decide this, case by case, based on the accuracy of the geometry, and, ideally, a sensitivity analysis should be conducted to test the influence of the number of faces on the FEA results (see (Bright and Rayfield 2011)).

**FIGURE 1:**
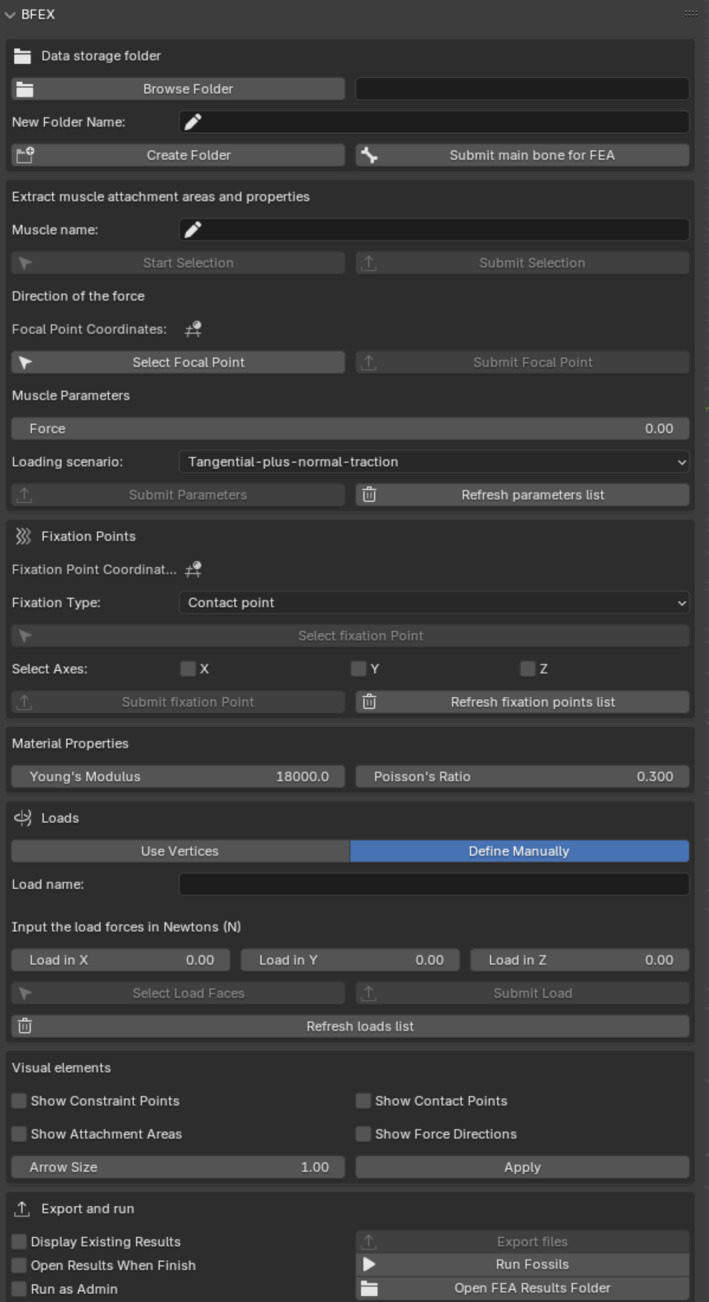
BFEX menu layout.

BFEX is divided into different sections, and not all of them are mandatory (Figure 1). The first section is the *Create folder* section, where the user can define the path to store the files and the name of the output folder. The second section is the *Main bone for FEA* section, where the user can select the main object to be analyzed. The third section is the *Muscle attachment areas* section, where the user can define the muscle attachment areas. The fourth section is the *Focal point* section, where the user can define the focal point of the muscle. The fifth section is the *Parameters* section, where the user can define the parameters of the muscle. The sixth section is the *Material properties* section, where the user can define the material properties of the bone. The seventh section is the *Loads* section, where the user can define the extrinsic forces applied to the model. The eighth section is the *Visual elements* section, where the user can add annotations to the model. The last section is the *Export and Run* section, where the user can create the FE model and run the FEA. In the initial section of the add‐on, users are prompted to specify a path to store the files and designate a name for the output folder to be created. Upon clicking the *Create folder* button, a new folder will be generated within the selected directory, along with a corresponding collection within the Blender scene. We recommend moving this collection to the top in the scene tree to improve the visualization of the sampled surfaces; otherwise, the attachment areas will be hidden below the main object (see Visual elements section below).

Next, users must identify the object (mesh) intended as the primary component for Finite Element Analysis (FEA) by selecting it within the scene and clicking the *Submit main bone for FEA* button.

Next step is to input the name of each muscle attachment area. By selecting the *Start selection* button, Blender will change to edit mode where the user will select the attachment area. Once selected, pressing the *Submit selection* button will create a new object containing all the selected faces in the previously created collection. The vertex groups section is located on the right panel of Blender, in the menu *Data/Object Data Properties*.

Then, it is necessary to determine the focal point of the muscle using an auxiliary object to determine the opposing attachment site. For example, if the user is analyzing a jaw, a mesh of the corresponding skull (or a proxy of it) will help to define the direction of the force. For this, the user should select the auxiliary object and, by pressing *Select focal point*, Blender will change to edit mode, allowing the user to select one vertex on the object to be used as the focal point to determine the direction of the force. Once selected, the user should save the coordinates by pressing *Submit focal point*. The next step is to define the magnitude of the muscle force (which can be previously obtained using the MyoGenerator add‐on; (Herbst et al. 2022)) and the loading scenario (tangential, uniform, directional, etc. For a full explanation and differences between loading scenarios see (Boman 2024 or Grosse et al. 2007)), and press *Submit parameters*. This button will store the name of the muscle, along with the defined parameters in a JSON dictionary in Blender. Also, it will create an object with one single vertex in the *Focal Points Collection*. In case of mistakes inputting the parameters, the user should delete the focal point object from the collection and press *Refresh Parameters list* to update the list of focal points stored. It is not necessary to delete the muscle attachment area since the submission of attachment areas is independent of the other parameters. It is only necessary to redefine the parameters and press *submit parameters* or delete the muscle attachment area and start again. This process should be repeated for each muscle that will be included in the model; this will create a section in the Python script containing the load parameters for the muscle‐driven simulation. For example:

*{*

*‘file’*: *f'{path}/pterygoideus_l.stl'*,

*‘force’*: *81.47000122070312*,

*‘focalpt’*: *[−8.104, −14.001*, *26.88]*,

*‘method’*: *‘T+N’*

*}*.

If the user wants to delete a contact or constraint point, it is necessary to delete the vertex group from the main object and press *Refresh fixation points* to update the list of stored fixation points.

The *Material Properties* section is where the user will input the values of the Young's modulus and the Poisson's ratio according to the values in the literature. The Young's modulus should be provided in megapascals (MPa). By default, a value of 18,000 MPa is given for the Young's modulus and a value of 0.3 for the Poisson's ratio. According to (Chatar et al. 2023), the density parameter is theoretically not required but should be provided. For this reason, the default value provided by the authors is automatically included in the generated Python file. The user may change these values if necessary by editing the Python file directly. The Python script will contain a section like the following:

*# material properties*.

*p[‘density’] = 1.662e‐9 # [T/mm¸s]*.

*p[‘Young’] = 18000.0 # [MPa]*.

*p[‘Poisson’] = 0.3 # [−]*.

The *Loads* section is used to define the extrinsic forces applied to the model. The user should define the name of the load, the vertex where it will be applied, and the magnitude of the force. This can be provided in two different ways. The first option (*Use vertices*) is used to define a vertex as the focal point to direct the force and another vertex where the force will be applied. The second option (*Define Manually*) works by selecting one vertex on the object and manually providing the magnitude and direction of the force applied in each axis. The Python script will contain a section like the following:

*p*[‘loads’] = [

{

“name”: “m1_r”,

“nodes”: [−29.853118896484375, 102.0189208984375, 70.42066192626953],

“values”: [0.0, 0.0, 4.35]

}].

It is important to notice that the user should apply all the modifiers and transformations to the main object before submitting the attachment areas, focal points, and fixation points. This is because if the topology of the mesh changes, *Fossils* will not be able to find the vertex coordinates on the mesh. Blender possesses a console where the users can track the result of each action done while creating the script, which can be seen in the *Scripting* workspace in Blender. It is recommended to check it to be sure an action was actually applied. This is especially important to check the content of the JSON dictionary with all the parameters to be exported.

**FIGURE 2:**
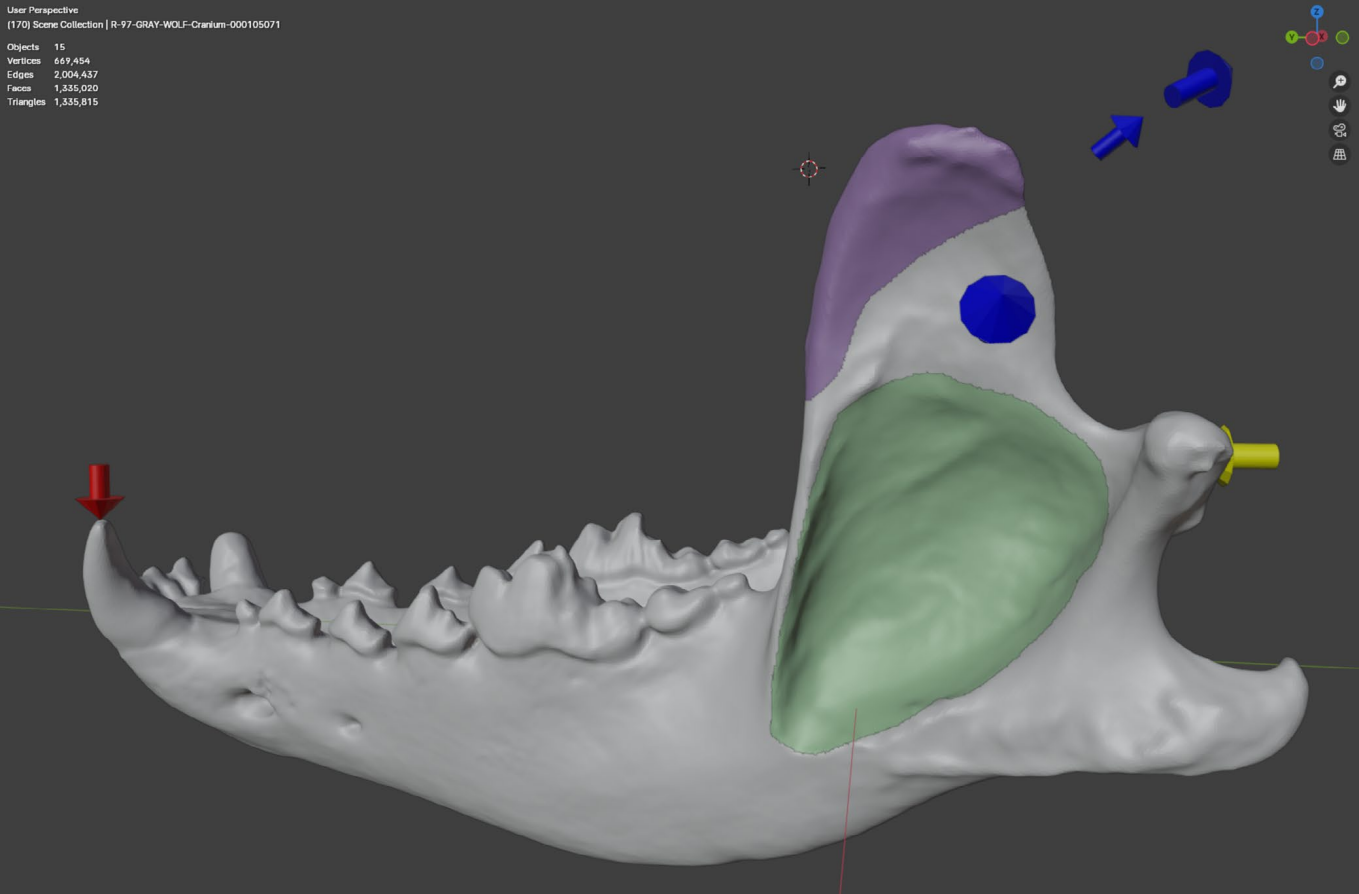
Visual elements generated by BFEX. Red arrows represent the contact points and loads, yellow arrows indicate constrained points, and blue arrows represent focal points (direction of the forces). Random colors are assigned to the muscle attachment areas.

The *Visual elements* section offers the flexibility to add annotations to the model (Figure 2) at any point. This can be very helpful not only to check the set parameters but also to create summary figures for publications. Red arrows represent the contact points (intrinsic) and loads (extrinsic); yellow arrows indicate constraint points, and blue arrows represent focal points or direction of the loads. Arrow sizes can be adjusted using the *arrow size* slider. Moreover, activating the *Show attachment areas* option randomizes the color of the meshes stored within the collection designated for muscle attachment areas. To observe these color changes effectively, ensure that the new collection is positioned at the top of the scene collection.

**FIGURE 3:**
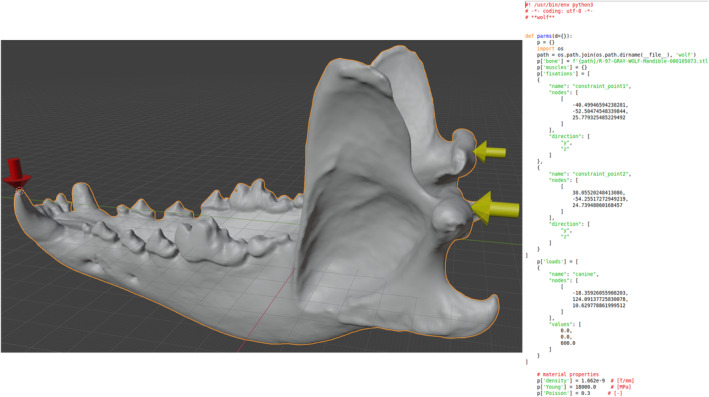
Example of the final results of the BFEX add‐on. The left panel shows the preview of a model where the tempomandibular joint is constrained in two points (yellow arrows). One load is applied to the canine (red arrow). The right panel shows the content of the generated Python file.

After defining all the parameters, in the *Export and Run* section the user can create the FE model using the *Export files* button. This action will create the Python file (Figure 3) containing all the defined parameters and a folder with one STL file for the main object and for each defined attachment area. It is possible to call *Fossils* directly from Blender to execute the model generated. This function will look for the *Fossils* binaries; the path should be provided in the preferences menu of the add‐on.

In the Github repository, we also provide a set of Python scripts to automate the process after exporting the files. These include (1) a script to run a batch of FEA with *Fossils*, which can be used, for example, to run several analyses at once (e.g., when conducting sensitivity analyses); (2) a script to export the Von Mises Stress values at each vertex (including their coordinates) as a CSV file and a summary of the results, including the mean, maximum, and minimum stress of the entire model. Also, the results are exported as .*vtk* files for easier visualization on other software such as ParaView. Those scripts are also available as executable files for Windows and Linux.

## 3 Conclusions

The use of Blender for scientific purposes has been increasing in recent years and will likely continue in the future thanks to its powerful 3D manipulation tools and ease of developing additional functions and add‐ons for specific purposes. Here we introduced a new tool for streamlining and making a time‐consuming and usually tedious process more user‐friendly. The use of BFEX will help to reduce the time spent in the preparation of the files for Finite Elements Analysis using *Fossils*. The add‐on is designed to guide the user through the process of creating the files needed by *Fossils* and offers the possibility of visualizing the model before exporting the files. BFEX will hopefully make FEA easier for new users and increase the usage of open‐source and free solutions, such as *Fossils*.

## Author Contributions

**E. Miguel Díaz de León‐Muñoz:** conceptualization (lead), data curation (lead), formal analysis (lead), investigation (lead), methodology (lead), project administration (lead), resources (lead), software (lead), supervision (lead), validation (equal), visualization (equal), writing – original draft (lead), writing – review and editing (lead). **Romain Boman:** software (supporting), validation (supporting), visualization (supporting), writing – original draft (supporting), writing – review and editing (supporting). **Gabriel S. Ferreira:** supervision (lead), validation (supporting), visualization (supporting), writing – original draft (equal), writing – review and editing (equal).

## Conflicts of Interest

The authors declare no conflicts of interest.

## Data Availability

Fossils can be found in the Fossils Repository (https://gitlab.uliege.be/rboman/fossils/). The software Blender is available at the Blender website (https://www.blender.org/). The BFEX add‐on can be accessed through Zenodo (https://doi.org/10.5281/zenodo.13908745). Future updates and developments for BFEX will be released on the Github repository: BFEX on Github (https://github.com/MiguelDLM/BFEX).

## References

[ece371093-bib-0001] Blender Development Team . 2024. Blender 4.1 Reference Manual. Blender Foundation. http://www.blender.org.

[ece371093-bib-0002] Boman, R. 2024. Fossils Documentation Version 1.3. https://gitlab.uliege.be/rboman/fossils/‐/wikis/home.

[ece371093-bib-0003] Bright, J. A. 2014. “A Review of Paleontological Finite Element Models and Their Validity.” Journal of Paleontology 88, no. 4: 760–769. 10.1666/13-090.

[ece371093-bib-0004] Bright, J. A. , and E. J. Rayfield . 2011. “The Response of Cranial Biomechanical Finite Element Models to Variations in Mesh Density.” Anatomical Record 294, no. 4: 610–620. 10.1002/ar.21358. 21370496

[ece371093-bib-0005] Buser, T. J. , O. F. Boyd , A. Cortes , et al. 2020. “The Natural Historian's Guide to the CT Galaxy: Step‐By‐Step Instructions for Preparing and Analyzing Computed Tomographic (CT) Data Using Cross‐Platform, Open Access Software.” Integrative Organismal Biology 2, no. 1: 25. 10.1093/iob/obaa009. PMC7671151 33791553

[ece371093-bib-0006] Chatar, N. , R. Boman , V. F. Gaudichon , J. A. MacLaren , and V. Fischer . 2023. “Fossils: A New, Fast and Open‐Source Protocol to Simulate Muscle‐Driven Biomechanical Loading of Bone.” Methods in Ecology and Evolution 14, no. 3: 848–859. 10.1111/2041-210X.14051.

[ece371093-bib-0007] Cignoni, P. , M. Callieri , M. Corsini , M. Dellepiane , F. Ganovelli , and G. Ranzuglia . 2008. “MeshLab: An Open‐Source Mesh Processing Tool.” In Eurographics Italian Chapter Conference, Edited by Vittorio Scarano, Rosario De Chiara, and Ugo Erra. EurographicsAssociation. 10.2312/LocalChapterEvents/ItalChap/ItalianChapConf2008/129-136.

[ece371093-bib-0008] Cunningham, J. A. , I. A. Rahman , S. Lautenschlager , E. J. Rayfield , and P. C. J. Donoghue . 2014. “A Virtual World of Paleontology.” Trends in Ecology & Evolution 29, no. 6: 347–357. 10.1016/j.tree.2014.04.004. 24821516

[ece371093-bib-0009] Díaz de León Muñoz, E. M . 2017. Manual de Fotogrametría en Paleontología. Universidad de Guadalajara.

[ece371093-bib-0010] DeVries, R. P. , P. C. Sereno , D. Vidal , and S. L. Baumgart . 2022. “Reproducible Digital Restoration of Fossils Using Blender.” Frontiers in Earth Science 10: 1–13. 10.3389/feart.2022.833379.

[ece371093-bib-0011] Garwood, R. , and J. Dunlop . 2014. “The Walking Dead: Blender as a Tool for Paleontologists With a Case Study on Extinct Arachnids.” Journal of Paleontology 88, no. 4: 735–746. 10.1666/13-088.

[ece371093-bib-0012] Grosse, I. R. , E. R. Dumont , C. Coletta , and A. Tolleson . 2007. “Techniques for Modeling Muscle‐Induced Forces in Finite Element Models of Skeletal Structures.” Anatomical Record 290, no. 9: 1069–1088. 10.1002/ar.20568. 17721980

[ece371093-bib-0013] Herbst, E. C. , L. E. Meade , S. Lautenschlager , N. Fioritti , and T. M. Scheyer . 2022. “A Toolbox For the Retrodeformation and Muscle Reconstruction of Fossil Specimens in Blender.” Royal Society Open Science 9, no. 8: 220519. 10.1098/rsos.220519. 36039284 PMC9399692

[ece371093-bib-0014] Lautenschlager, S. 2015. “Estimating Cranial Musculoskeletal Constraints in Theropod Dinosaurs.” Royal Society Open Science 2, no. 11: 150495. 10.1098/rsos.150495. 26716007 PMC4680622

[ece371093-bib-0015] Maas, S. A. , B. J. Ellis , G. A. Ateshian , and J. A. Weiss . 2012. “FEBio: Finite Elements for Biomechanics.” Journal of Biomechanical Engineering 134, no. 1: 011005. 10.1115/1.4005694. 22482660 PMC3705975

[ece371093-bib-0016] Marcé‐Nogué, J. , S. de Esteban‐Trivigno , C. Escrig , and L. Gil . 2016. “Accounting for Differences in Element Size and Homogeneity When Comparing Finite Element Models: Armadillos as a Case Study.” Palaeontologia Electronica 19, no. 2: 1–22. 10.26879/609.

[ece371093-bib-0017] Marcé‐Nogué, J. , J. Fortuny , L. Gil , and M. Sanchez . 2015. “Improving Mesh Generation in Finite Element Analysis for Functional Morphology Approaches.” Spanish Journal of Palaeontology 30, no. 1: 117–132. 10.7203/sjp.30.1.17227.

[ece371093-bib-0018] Rayfield, E. J. 2007. “Finite Element Analysis and Understanding the Biomechanics and Evolution of Living and Fossil Organisms.” Annual Review of Earth and Planetary Sciences 35: 541–576.

[ece371093-bib-0019] Sutton, M. , I. Rahman , and R. Garwood . 2016. “Virtual Paleontology—An Overview.” Paleontological Society Papers 22: 1–20. 10.1017/scs.2017.5.

[ece371093-bib-0020] Zhang, C. , and A. M. Maga . 2023. “An Open‐Source Photogrammetry Workflow for Reconstructing 3D Models.” Integrative Organismal Biology 5, no. 1: obad024. 10.1093/iob/obad024. 37465202 PMC10350669

[ece371093-bib-0021] Zienkiewicz, O. C. , and R. L. Taylor . 2005. The Finite Element Method for Solid and Structural Mechanics. Elsevier.

[ece371093-bib-0022] Zienkiewicz, O. C. , and P. B. Morice . 1971. The Finite Element Method in Engineering Science. McGraw‐Hill Vol. 1.

